# Erchen decoction for hyperlipemia: Protocol for a systematic review and meta-analysis

**DOI:** 10.1097/MD.0000000000022374

**Published:** 2020-10-16

**Authors:** Huan Luo, Min Xiong, Wenyu Zhu, Tao Shen

**Affiliations:** College of basic medicine, Chengdu university of Traditional Chinese Medicine, Chengdu, Sichuan 611137, China.

**Keywords:** hyperlipidemia, atherosclerosis, protocol, systematic review, Erchen decoction

## Abstract

**Background:**

Hyperlipidemia is a metabolic disease characterized by elevated levels of blood lipids and lipoproteins and a major pathogenic factor of atherosclerosis. In China, Erchen decoction (ECD) has been widely used to treat hyperlipidemia. However, there is no systematic review found. In order to evaluate the efficacy and safety of ECD in the treatment of hyperlipidemia, we need to conduct a meta-analysis and systematic evaluation.

**Methods:**

We will enroll the randomized controlled trials (RCTs) evaluating the effectiveness and safety of ECD in the treatment of hyperlipidemia. Data come mainly from 4 Chinese databases (China national knowledge infrastructure, Wanfang, Chinese biomedical literature database, and VIP Database) and 4 English databases (Pubmed, Embase excerpt medica database, Cochrane Library, and Web of science). The enrollment of RCTs is from the starting date of database establishment till September 30, 2020. Low density lipoprotein cholesterol is considered as the main indicator of the dyslipidemia, while the serum concentrations of total cholesterol, triglyceride, high density lipoprotein cholesterol, apolipoprotein A, and apolipoprotein B are regarded as the secondary indicators. There are Safety indicators including liver enzyme, kidney function and fasting blood glucose. The work such as selection of literature, data collection, quality evaluation of included literature, and assessment of publication bias will be conducted by 2 independent researchers. Meta-analysis will be performed by RevMan 5.0 software.

**Results:**

This study will provide high-quality evidence for the effectiveness and safety of ECD in the treatment of hyperlipidemia.

**Conclusion:**

The results of the study will help us determine whether ECD can effectively treat hyperlipidemia.

**Ethics and dissemination:**

This study does not require ethical approval. We will disseminate our findings by publishing results in a peer-reviewed journal.

**OSF registration number:**

DOI 10.17605/OSF.IO/GZ69F

## 1 Introduction

Hyperlipidemia is a chronic metabolic disease characterized by elevated levels of serum cholesterol, triglyceride or low-density lipoprotein cholesterol (LDL) and decreased levels of high-density lipoprotein cholesterol.^[1]^ Chinese data from the International Study on Dyslipidemia (DYSIS) and the Chinese Study on Statin-enhanced Lipid-lowering of Acute Coronary Syndrome (CHILLAS study) also showed that the LDL-C compliance rate in Chinese patients was still at a low level.^[2]^ According to statistics, the prevalence of dyslipidemia in China is about 20% of the total population. In paiticularly, the prevalence of low high-density lipoproteinemia was 44.8%; Triglyceridemia was 11.3%; Hypercholesterolemia was 3.3%; High low-density lipoproteinemia was2.1%.^[3]^ The studies have shown that dyslipidemia can cause chronic inflammatory reactions in the body, and the release of inflammatory factors can increase the risk of atherosclerosis by damaging vascular endothelial cells and vascular walls.^[4]^ Among them, LDL-C is a basic factor leading to atherosclerotic lesions.^[5–8]^ Therefore, lipid-lowering therapy is of great significance. as we know, statins are lipid-lowering drugs widely used in clinical practice with definite efficacy, but adverse reactions of the drug, such as liver and kidney damage and rhabdomyolysis, have been also highly controversial.^[9]^ In this case, the lipid-lowering treatment of alternative medicine has attracted more people's attention. There are more studies which have shown that the prescription represented by Erchen decoction (ECD) which is composed of 4 traditional Chinese medicines, pinellia ternata, Orange peel, Poria cotta, and licorice has a laudable lipid-lowering effect.^[10–12]^ However, we have found no systematic study on the efficacy and safety of ECD lipid-lowering therapy. So we will systematically evaluate the efficacy and safety of ECD in treating hyperlipidemia by a meta-analysis method, which provide strong evidence-based medicine support for its clinical applications.

## 2 Methods

### 2.1 Protocol and registration

The protocol has been registered on the Open Science Framework (OSF) platform (https://osf.io/tahnd/), registration number: DOI 10.17605/OSF.IO/GZ69F. This protocol was drafted and reported in accordance with the Preferred Reporting Items for Systematic Reviews and Meta-Analyses Protocols (guidelines.^[13]^ The final report will comply with the recommendations of the preferred reporting items for systematic review and meta-analysis protocols extension statement for reporting of systematic reviews incorporating meta-analyses of healthcare interventions.^[14]^

### 2.2 Ethics

We will not need individual data of each patient in the research as this is a systematic review. Therefore, institutional review board approval and ethics committee is not needed. Our purpose is to publish the results in a peer-reviewed journal. The final results of the review will provide information about the safety and efficacy of ECD and its modified forms in the treatment of hyperlipemia (HLP) to help clinicians make decisions on clinical practice.

### 2.3 Eligibility criteria

The participant (P), intervention (I), comparator (C), outcome (O), and study design (S) are the 5 main factors determining the inclusion and exclusion criteria of this research.

#### 2.3.1 Type of study design

We will exclude quasi-RCT, non-RCT, observation group combination other drugs, animal experiments, control groups not match and full texts not available by reading the title, abstract and related quotations Information screening. Then we can eliminate incomplete experimental data and experimental design schemes not rigorous, no clear diagnostic criteria by reading and understanding the full paper, and finally randomized controlled trials were included this research.

#### 2.3.2 Type of participant

Adult participants (older than 18 years of age) with HLP and no other illness will be enrolled. The diagnosis of HLP can be established if the patient’ blood lipids remain high 2 to 4 weeks later after his initial visit.^[15,16]^ No gender, race, nationality and comorbidity are limited.

#### 2.3.3 Type of interventions

The patients in the treatment groups will be given ECD or modified ECD as a monotherapy or in combination with conventional therapy. ECD consisting of 4 herbs: pinellia ternata, Orange peel, Poria cotta, and licorice. According to “Jun-Chen-Zuo-Shi” principle of Chinese herbal formula, Orange peel and pinellia ternata are “Jun” herb, and Poria cotta is “Chen” herb, both of which are the core of ECD. Therefore, modified ECD should include pinellia ternata, Orange peel, Poria cotta basically. The number of modified herbs will not exceed 3(n≤3). Patients of control group will be treated with conventional therapy with placebo, stains and fibrates. In addition, the 2 groups have not taken any drugs that possibly interfered with the outcome indicators. The follow-up time was ≥4 weeks.

#### 2.3.4 Type of outcomes

##### 2.3.4.1 (1) Primary outcomes

According to the “Clinical Research Guidelines for New Chinese Medicines” and the new AACE/ACE guidelines, LDL-C is determined as the main indicator. If LDL-C decreases by ≥20%, it is considered to have a significant effect^[15,17]^; if LDL-C decreases by 10% to 20%, it is considered to be effective; invalid, if the level of LDL-C decline does not meet the above criteria.

##### 2.3.4.2 (2) Secondary outcomes

According to the “Clinical Research Guidelines for New Chinese Medicines,”^[15]^ the secondary indicators include serum concentration of TC, TG, high-density lipoprotein cholesterol, apolipoprotein A, and apolipoprotein B.

##### 2.3.4.3 (3) Safety outcomes

Safety indicators consist of liver enzyme, kidney function and fasting blood glucose.

### 2.4 Literature retrieval strategy

Computer search of PubMed, the Cochrane Library, excerpt medica database, China National Knowledge Infrastructure, China Biomedicine, Chinese Scientific Journals Database (VIP), Wanfang Database for published information about RCTs of ECD for the treatment of hyperlipidemia. The time limit for literature search is from the establishment of each database to September 30, 2020. The language is limited to English and Chinese. In addition, the World Health Organization International Clinical Trials Registration Platform and Clinical Trials website (Clinical Trials.gov) will be searched for ongoing trials related to the disease and RCTs in China. The search method uses a combination of free words and medical subject terms, including: “hyperlipidemia,” “lipid reduction,” “ dyslipidemia,” “spleen and stomach,” “Chinese medicine,” “erchen decoction,” and so on. Chinese database search will use the following terms: “gaozhixuezheng,” “jiangzhi,” “xuezhiyichang,” “piwei,” “zhongyao,” “erchentang,” and so on. Taking PubMed as an example, the initial search strategy is shown in Table 1, which will be adjusted according to the specific database.

**Table 1:**
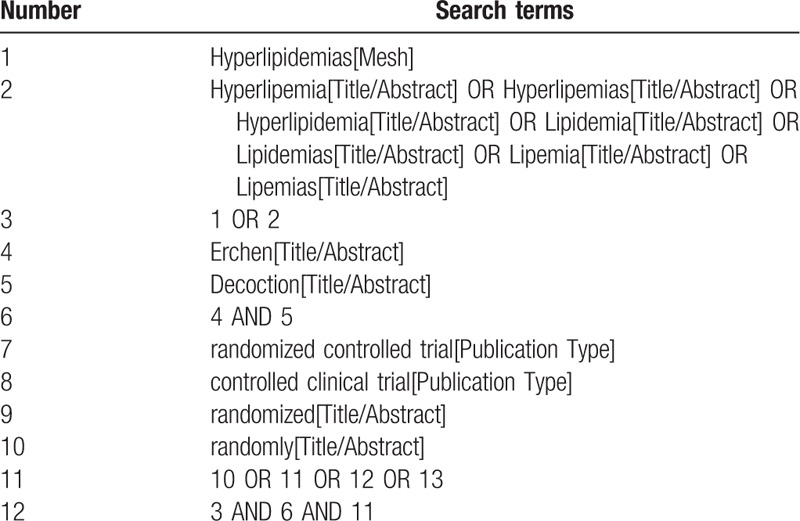
Search strategy of the PubMed.

### 2.5 Data collection and analysis

#### 2.5.1 Literature selection and data extraction

**Figure 1:**
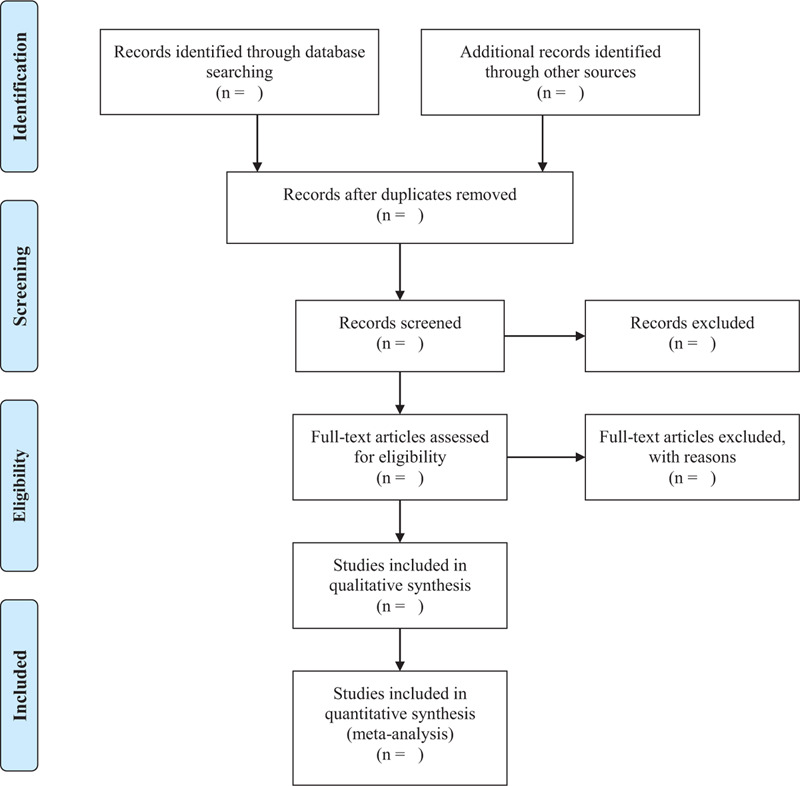
PRISMA flow diagram of the study selection process. PRISMA = preferred reporting items for systematic review and meta-analysis.

As shown in Figure 1, 2 researchers (Huan Luo and Min Xiong) will screen the documents according to the inclusion and exclusion criteria:

(1) Import the retrieved documents into Endnote X9 software for review, then remove duplicate references;
(2) By preliminary screening abstract, we exclude documents which obviously do not meet the inclusion criteria;
(3) Download and read the full paper for follow-up examination;
(4) After the final inclusion, we will use the pre-designed data extraction table for data extraction and cross-check the results;
(5) If there is any objection, the third researcher (Wenyu Zhu) will be asked to assist in the judgment.

The main content of data extraction includes: basic information of literature (title, journal, author, publication date), basic information of the research object (sample size, gender, average age, intervention, and course of treatment), and result data (numbers of response events, non-response events, dropouts, time points, mean, SD, follow-up time and adverse events). If the required information is missing or incomplete, we will contact the relevant email address of the corresponding author or first author of the original document. If the relevant data can not be obtained, the record is excluded. At the same time, the key factors of bias risk assessment are extracted.

#### 2.5.2 Risk of bias assessment

The method quality of systematic review reflects the risk of deviation or validity in its process and results. The quality of the method will be evaluated according to Cochrane Manual 5.2.0.^[18]^ Two well-trained researchers (Huan Luo and Min Xiong) independently assessed the risk of bias in the study. The evaluation content includes generation of random sequences, randomization concealment, the implementation of blinded subjects and researchers, the implementation of blind methods for outcome evaluators, the integrity of outcome data, selective reporting, and other biases. Each item should be judged as 3 “low risk” levels bias, “high-risk bias,” and “unclear” follow quality classification standards. For each item, if it is satisfactory, it means “low risk deviation,” if not, it means “high risk deviation.” When there is not enough information in the literature to make a clear judgment on the corresponding item, it means “unclear.” If there is any dispute, we will submit it to the corresponding author (Tao Shen) for arbitration.

#### 2.5.3 Data synthesis

Meta-analysis will be performed by RevMan 5.0 software (Version 5.3, Copenhagen: The Nordic Cochrane Center, 2014) provided by the Cochrane Collaboration. While there is statistical homogeneity between each study (*I*^2^ < 50%), the fixed effect model is used. When the heterogeneity is significant (*I*^2^ ≥ 50%) between the results of each study, the sub-layer analysis is performed to find the source of heterogeneity. A fixed effect model is used for meta-analysis when there is sufficient similarity between the results of the subgroups(*I*^2^ < 50%). However, a random effect model is used for meta-analysis if the heterogeneity between the results of the subgroups is significant (*I*^2^ ≥ 50%). Qualitative heterogeneity is used when heterogeneity is too large or the source of heterogeneity is unknown. Meta-regression analysis can be performed if there are many influencing factors and it is not appropriate to use the stratification method.

#### 2.5.4 Assessment of heterogeneity

Before the combination of effect size, the heterogeneity of the included literature is tested using Stata 13.0. When inter-study heterogeneity exists, the random effect model will be used. For comparison of each pair, Heterogeneity is determined by heterogeneity test and expressed by *I*^2^ value. When *I*^2^ ≥ 50%, the heterogeneity is large. When 25% < *I*^2^ < 50%, we think it is moderate. When *I*^2^ < 25%, the heterogeneity is considered small.

#### 2.5.5 Sensitivity analysis

Sensitivity analysis is based on sample size, missing data results, and methodological quality. Sensitivity analysis will be put into effect to examine the robustness of the pooled results in case of sufficient data by determining the effects of excluding studies with high risks of bias or with missing data, and outliers.

#### 2.5.6 subgroup analysis

Subgroup analysis is to explore the source of heterogeneity. When more than 10 studies are included, subgroup analyses will be performed based on different participants, gender, duration of disease, interventions, and dose. We can better explore the source of heterogeneity as investigator by this method.

#### 2.5.7 Assessment of reporting bias

If 10 or more papers are conducted, a comparison-adjusted funnel plot is developed to using Stata to evaluate the presence of small sample effects or publication bias in the intervention. Descriptive analysis will be carried out by the method of the symmetry of funnel plot. If there is asymmetric or no inverted funnel in the plot, it is deemed that there may be publication bias. It is possibly connected with the difficulty in the publication of the literature with negative results and the low quality of the inclusion methods.

#### 2.5.8 Grading the quality of the evidence

To grade evidence quality and understand the actual situation of evidence rating thereby analyzing possible questions, the Grading of Recommendations Assessment, Development and Evaluation (GRADE) system will be used to evaluate the quality of evidence.^[19]^ On account of the risk of bias, imprecision, inconsistency, indirection, and publication bias, GRADE grades evidence quality into 4 levels: high, medium, low and very low.

## 3 Discussions

Hyperlipidemia is a common chronic disease in modern society and also a major risk factor for various cardiovascular diseases such as Atherosclerosis. According to the cause of disease, it can be divided into primary and secondary, and the primary cause related to factors such as genetics, diet, nutrition, drugs, and so on. At present, its overall control is not ideal.^[20]^ It is reported that some drugs which is used to reduce cardiovascular risk factors may have adverse effects.^[21]^ For example, some patients show intolerance to statins,^[22]^ so it is always controversial to use statins as the first lipid-lowering drugs in clinical practice.^[23]^ More studies have shown that ECD can not only control blood lipid levels, but also effectively relieve symptoms such as dizziness and chest tightness in patients with hyperlipidemia. However, there is no systematic review and meta-analysis to evaluate its efficacy and safety at present. Therefore, it is necessary to provide compelling evidence for the advantages of ECD in hyperlipidemia through a high-quality systematic review and meta-analysis. In addition, there may still be potential shortcomings in this study. First of all, the form of research in Chinese and English will likely increase the bias of research. Secondly, age, gender, race, drug formulation, dosage, and course of treatment can lead to the risk of heterogeneity. finally, the study may involve a small number of clinical trials, leading to a high risk of bias.

## Author contributions

Huan Luo and Min Xiong made the same contribution to the research and design, and wrote the original draft of the protocol. Huan Luo has developed a search strategy. Huan Luo, Min Xiong and Wenyu Zhu will conduct literature retrieval and collation. Huan Luo, Min Xiong and Tao Shen will evaluate the risk of bias in the literature. Data analysis and article writing will be done by Huan Luo, Min Xiong. Tao Shen, as the corresponding author, will be responsible for overseeing every process of the audit review to control the quality of the study. All the authors have approved the publication of the protocol.

**Conceptualization:** Huan Luo.

**Data curation:** Huan Luo, Min Xiong.

**Formal analysis:** Huan Luo, Min Xiong, Wen-yu Zhu.

**Funding acquisition:** Tao Shen.

**Investigation:** Huan Luo, Min Xiong, Wen-yu Zhu.

**Methodology:** Huan Luo, Min Xiong.

**Project administration:** Tao Shen.

**Resources:** Tao Shen.

**Software:** Tao Shen.

**Supervision:** Wen-yu Zhu.

**Visualization:** Wen-yu Zhu.

**Writing – original draft:** Huan Luo, Min Xiong.

**Writing – review & editing:** Tao Shen.
